# Hybrid approach to hemobilia: endoscopic and endovascular management of a ruptured hepatic artery pseudoaneurysm

**DOI:** 10.1055/a-2578-2987

**Published:** 2025-04-28

**Authors:** Takamitsu Tanaka, Reiko Yamada, Kenji Nose, Yoshifumi Nakamura, Tetsuro Miwata, Masashi Fujimori, Hayato Nakagawa

**Affiliations:** 1 220937 Department of Gastroenterology and Hepatology, Mie University Hospital, Tsu, Japan; 2 220937 Department of Radiology, Mie University Hospital, Tsu, Japan

Hemobilia caused by hepatic pseudoaneurysms is rare but can be fatal 1 2 3 . We report a case in which simultaneous endoscopic retrograde cholangiography (ERC) with endovascular treatment resulted in the successful embolization of a ruptured hepatic pseudoaneurysm.

A 70-year-old man was admitted to our hospital with recurrent hemobilia of unknown etiology. He had previously undergone chemoradiotherapy for pancreatic head cancer. Although he presented with anemia, blood transfusions were difficult because of his constitution. Upon admission, bleeding had temporarily ceased after the placement of a fully covered self-expandable metal stent (FCSEMS) via ERC; however, contrast-enhanced computed tomography revealed the FCSEMS had become displaced into the bowel and a right hepatic artery (RHA) pseudoaneurysm was present on the common bile duct (CBD) wall ( Fig. 1 ). Coil embolization of the pseudoaneurysm was planned; however, owing to the high risk of rupture during endovascular treatment, the procedure was performed alongside biliary balloon implantation via ERC for rapid hemostasis ( Video 1 ).

A ruptured right hepatic artery aneurysm forming a fistula with the common bile duct is successfully managed using a combined approach of endovascular treatment and endoscopic retrograde cholangiography, which facilitated prompt hemostasis and guidewire control, resulting in successful coil embolization of the pseudoaneurysm.Video 1

**Fig. 1:**
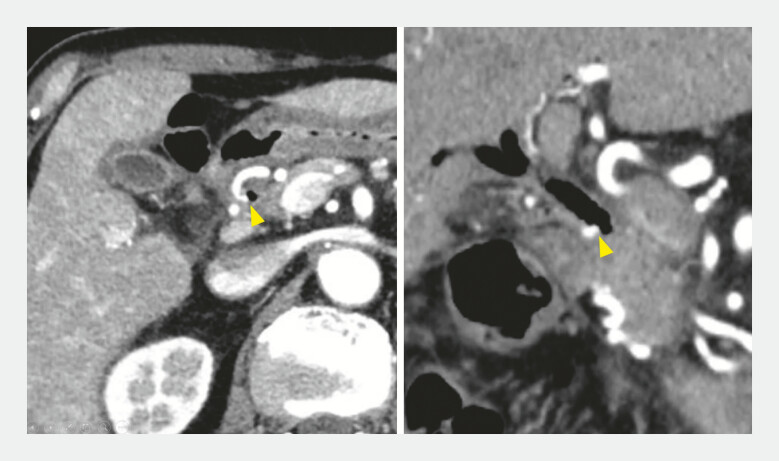
Contrast-enhanced computed tomography scan showing the displaced fully covered self-expandable metal stent in the bowel and the right hepatic artery pseudoaneurysm on the common bile duct wall (arrowhead).

The endovascular treatment was performed with the patient under general anesthesia in a hybrid operating room. An 8-mm balloon catheter (REN; Kaneka, Osaka, Japan) was placed in the CBD to mitigate hemobilia in case of aneurysm rupture ( Fig. 2 ). While the distal vessel of the pseudoaneurysm was being sought, the microguidewire was advanced out of the pseudoaneurysm into the CBD. Hemobilia was observed endoscopically, and the balloon was expanded. Prompt hemostasis was achieved, and the dilated balloon pushed the guidewire back from the CBD into the distal vessel ( Fig. 3 ). Coil embolization was performed alongside balloon dilation, followed by placement of an FCSEMS (Hanarostent, 8 × 60 mm; Boston Scientific, Natick, Massachusetts, USA). Subsequently, the patient had no further recurrent hemobilia ( Fig. 4 ).

**Fig. 2:**
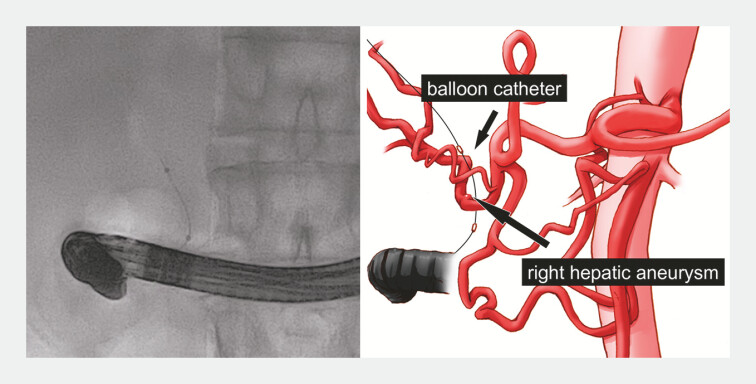
Fluoroscopic and schematic images showing the 8-mm balloon catheter positioned in the common bile duct to prevent hemobilia if the aneurysm were to rupture.

**Fig. 3:**
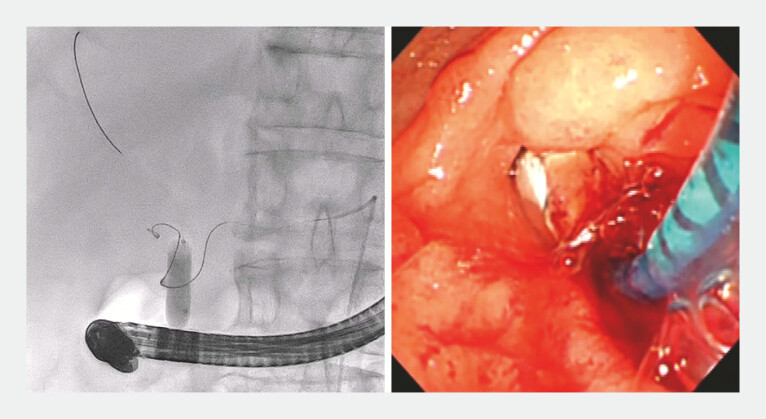
Fluoroscopic and endoscopic images showing prompt hemostasis being achieved and the dilated balloon pushing the guidewire back from the common bile duct into the distal vessel.

**Fig. 4:**
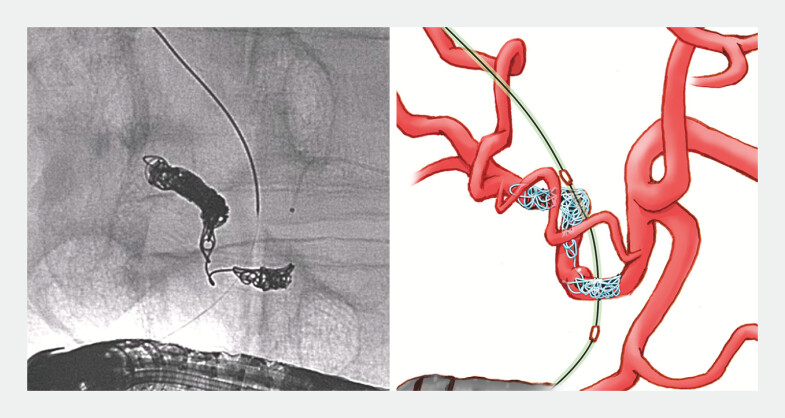
Fluoroscopic and schematic images of the successful coil embolization procedure.

A ruptured RHA aneurysm forming a fistula into the CBD was successfully managed using a combined approach of endovascular treatment and ERC, which facilitated prompt hemostasis and guidewire control, resulting in successful coil embolization of the pseudoaneurysm.

Endoscopy_UCTN_Code_TTT_1AR_2AZ

## Notes

Endoscopy E-Videos https://eref.thieme.de/e-videos
*E-Videos* is an open access online section of the journal *Endoscopy* , reporting on interesting cases and new techniques in gastroenterological endoscopy. All papers include a high-quality video and are published with a Creative Commons CC-BY license. Endoscopy E-Videos qualify for HINARI discounts and waivers and eligibility is automatically checked during the submission process. We grant 100% waivers to articles whose corresponding authors are based in Group A countries and 50% waivers to those who are based in Group B countries as classified by Research4Life (see: https://www.research4life.org/access/eligibility/ ). This section has its own submission website at https://mc.manuscriptcentral.com/e-videos .

## References

[1] Walter JF Paaso BT Cannon WB Successful transcatheter embolic control of massive hemobilia secondary to liver biopsy AJR AM J Roentgenol 1976 127 847 849 973674 10.2214/ajr.127.5.847

[2] Zhornitskiy A Berry R Han JY Hemobilia: Historical overview, clinical update, and current practices Liver Int 2019 39 1378 1388 10.1111/liv.14111 30932305

[3] Staszak JK Buechner D Helmick RA Cholecystitis and hemobilia J Surg Case Rep 2019 2019 rjz350 10.1093/jscr/rjz350 PMC6911657 31857891

